# No Smallpox Epidemic at Niagara Falls

**Published:** 1913-03

**Authors:** 


No Smallpox Epidemic at Niagara Falls. Dr. E. E. Gillick, Health Officer, denies the press report that there was to be a wholesale vaccination or that there is any need of it. He thinks the rumor may have arisen from an agent's attempt to sell the city a large supply of vaccine.

